# Innovation Strategies and Health System Guiding Principles to Address Equity and Sustainability in Responsible Innovation in Health: Comment on "What Health System Challenges Should Responsible Innovation in Health Address? Insights From an International Scoping Review"

**DOI:** 10.15171/ijhpm.2019.50

**Published:** 2019-07-06

**Authors:** Sandra C. Buttigieg

**Affiliations:** ^1^Department of Health Services Management, Faculty of Health Sciences, University of Malta, Msida, Malta.; ^2^Health Services Management Centre, School of Social Policy, College of Social Sciences, University of Birmingham, Birmingham, UK.

**Keywords:** Equity, Health System Principles, Innovation Strategies, Political Systems, Sustainability

## Abstract

The insights from an international scoping review provided by Lehoux et al challenge health policy-makers, entrepreneurs/innovators and users of healthcare, worldwide, to be aware of equity and sustainability challenges at system-level when appraising responsible innovation in health (RIH) – purposefully designed to better support health systems.The authors manage to extract no less than 1391 health system challenges with those mostly cited pertaining to service delivery, human resources, leadership and governance. Countries were classified according to the Human Development Index (HDI), while the authors decided not to classify according to the types of health systems justifying this on the basis that the articles reviewed studied a specific setting within a broader national or regional health system. The article presents highly powerful and discerning viewpoints, indeed providing numerous standpoints, yet in a comprehensive manner, thereby putting structure to a somewhat highly complex and multidimensional subject. This commentary brings forth several considerations that are perceived on reading this article. First, although innovation strategies are important for the dynamicity of health systems, one should discuss whether or not RIH can adequately address equity and sustainability on a global scale. Secondly, RIH across countries should also be debated in the context of the principles garnered by the type of health system, thereby identifying whether or not the prevailing political goals support equity and sustainability, and whether or not policy-makers are adequately supported to translate system-level demand signals into innovation development opportunities. As key messages, the commentary reiterates the emphasis made by the authors of the need for international policy-oriented fora as learning vehicles on RIH that also address system-level challenges, albeit the need to acknowledge cultural differences. In addition, the public has not only the right for transparency on how equity and sustainability challenges are addressed in innovation decisions, but also the responsibilities to contribute to overcome these challenges.

The insights from an international scoping review provided by Lehoux et alchallenge health policy-makers, entrepreneurs/innovators and users of healthcare, worldwide, to be aware of equity and sustainability challenges at system-level when appraising responsible innovation in health (RIH) – purposefully designed to better support health systems.^1^ Equity in health is “the absence of systematic disparities in health (or in the major social determinants of health) between groups with different levels of underlying social advantage/disadvantage—that is, wealth, power, or prestige” (p. 254).^2^ Although there is no widely-used definition of sustainability in healthcare, in this commentary, I will refer to it as a key task for health policy-makers to withstand social, financial, and ecological pressures and challenges.^3^

The authors highlight interesting research gaps that inspired them to conduct the international scoping review, namely that “few attempts have been made to articulate what system-level challenges RIH should seek to address” and “one may wonder why health services and policy researchers have not yet sought to synthesize what is known about system-level challenges that innovations should attend to in the first place.” Specifically, the authors frame the discourse on innovation against the population health goals of equity and sustainability. Indeed, the review enabled the authors to fill these gaps by putting structure to the complex and somewhat fragmented issues surrounding health-system’ demands for innovations, and utilizing van Omen et al’s analytical framework^4^ to argue that innovations may affect several health system components simultaneously, and that some innovations may alleviate, while others exacerbate equity and sustainability challenges. What comes out very clearly is that it is somewhat difficult for health innovators to satisfy RIH’s definition in its entirety and therefore the “ethical, economic, social and environmental principles, values and requirements when they design, finance, produce, distribute, use and discard sociotechnical solutions to address the needs and challenges of health systems in a sustainable way.”^5^ Likewise, it is taxing for policy-makers to strain health systems’ financing and governance so as to enable diffusion of medical technologies. In other words, health policy-makers and other stakeholders experience ethical dilemmas on a daily basis in their bid to keep up with medical innovations, yet strive to reach the optimal trade-off between competing demands, equity and sustainability. In this commentary, I will refer to healthcare in general with examples cutting across different specializations and disciplines, since all these contribute in one way or other to population health.

This commentary brings forth the following considerations on this article.

## Innovation Strategies

Lehoux et almanage to extract no less than 1391 health system challenges with those mostly cited pertaining to service delivery, human resources, leadership and governance.^1^ This fits within the analytical framework adapted from Olmen et al.^2^ The findings support the innovation strategy of building capacity, resources, knowledge, skills etc as part of an innovation-friendly infrastructure. The challenge that arises is that not all countries, in particular those classified as the lower income ones, can afford to build up this infrastructure that is needed to support the innovation strategy. The limited governance structures found in most low-income countries may also be the cause for the failure in implementing any form of innovation strategy, even if resources are made available by richer countries.^6,7^ Therefore, the attainment of global equity and sustainability is near to impossible to achieve because of the multifactorial conditions that need to be in place for the innovation strategies to work. Rightly so, the authors classify countries according to the Human Development Index (HDI) – a proxy for population health as it considers life expectancy at birth, mean years of schooling and expected years of schooling, and gross national income per capita. The discourse on equity is of an ethical nature, and based on the principles of distributive justice in a bid to allocate scarce resources in a fair and just manner. Interestingly, the authors acknowledge that Brazil, India, China, and South Africa are active in health innovation development, even though they are outside the Organization for Economic Cooperation and Development, which provides unity among member countries to share common eco-social problems, as well as collaborate on finding solutions. While this is promising in that the ‘poorer’ countries are important players in innovation, a comparison of the historical medical spending patterns among the BRICS (Brazil, Russia, India, China, and South Africa) and G7 countries reveals that the egalitarian principle of global equity is not supported in view of the gross global wealth inequality in relation to the size of the population served – which is reflected in health spending.^8^ Indeed, the BRICS’ populations’ actual health needs – reflected by the socio-economic determinants, are far greater than those of G7 countries, yet the their share of Total Health Expenditure is far less.^8^

Witty^9^ attempts to suggest new strategies for innovation in global health by taking the pharmaceutical perspective. He argues that since pharmaceutical industries are the ones driving crucial research into new vaccines and medicines to combat diseases that disproportionately affect developing countries, they need to be supported to secure financial returns that safeguards their investment in neglected and less lucrative diseases. Witty^9^ claims that “the public and private sectors must work together to develop a wide range of innovative tools, partnerships and approaches” (p. 118). Among these include an open innovation strategy that redesigns sharing intellectual property, resources, and data – and therefore introducing flexibility, easier accessibility to libraries and collections of molecular entities, as well as opportunities for external researchers to work alongside company scientists.

The crisis from emerging zoonosis like Ebola led to stakeholders worldwide to adopt a One Health approach.^10^ This applies to innovations, as the smart way of achieving equity and sustainability in addressing ‘wicked’ problems that have eluded policy-makers for so long is the transdisciplinary collaboration in ensuring sharing of new knowledge, skills, technologies and infrastructure.^11^ Wicked problems are challenging, multifaceted, and intractable healthcare issues that contribute disproportionately to reduced quality of life, chronic health conditions, and high healthcare utilization.^12^ Examples include stigmatized conditions, such as obesity, substance use disorders, and domestic violence, which could be addressed by innovative systems, quality improvement methodologies, health information technologies, and implementation science.^12^

This is in line with RIH which as cited by Lehoux et al is understood as a “collaborative endeavor wherein stakeholders are committed to clarify and meet a set of ethical, economic, social and environmental principles, values and requirements when they design, finance, produce, distribute, use and discard sociotechnical solutions to address the needs and challenges of health systems in a sustainable way.”

Other strategies suggested by Witty^9^ is redesigning financing schemes and pricing models to boost private sector investment in drug/vaccine research and development, as well as the dissemination, diffusion, adoption and implementation of existing technologies, therapies for developing countries.

Finally, adopting a disruptive innovation strategy is more likely to reach populations far and wide. Disruptive innovations in healthcare replace existing services by low-cost alternatives in what is deemed to be “Uber’s message for healthcare.”^13^ Examples include the various types of point-of-care testing at the emergency department, for example use of troponin testing for acute coronary syndrome and abdominal ultrasound for blunt abdominal trauma, that are now increasingly saving lives in emergency situations.^14^

## Health Systems’ Guiding Principles and Political Agenda Prevailing in Country 

Lehoux et al^1^ classified countries according to HDI, while justifying their decision not to classify them according to the types of health systems on the basis that the articles reviewed studied a specific setting within a broader national or regional health system. However, RIH across countries should also be discussed in the context of the principles garnered by the type of health system, thereby identifying whether or not the prevailing political goals support equity and sustainability, and whether or not policy-makers are adequately supported to translate system-level demand signals into innovation development opportunities. Interestingly, principles and values were scantly reported as system-level challenges for RIH across the spectrum of HDI countries and may be considered an important research gap to fill. The slow diffusion, adoption and implementation, as well as the attainment of equity and sustainability of RIH, may also be researched by reviewing the context in which they were borne, namely the guiding principles, environmental and operational characteristics of the healthcare system.^15^ Berwick identified politics and red tape as barriers to innovation.^16^ The guiding principles, namely commitment to solidarity, equity and universality are not given equal importance across health systems and therefore system-levels goals of RIH are not the same across countries. These differences may also influence the transfer of innovations across systems. For example, the rivate mixed health services model of the United States is based on the principles of high quality of care, advanced medical technology and best models of specialist care but less on solidarity and commitment to universal coverage. In contrast, the state as owner model (as in United Kingdom) or the state as guardian model (as in Germany) of health services are based on the principles of solidarity and universality.^15^ The latter however seems to be more successful at adopting a consumer-driven approach at innovation than the former.

## Conclusion

This commentary provides some insights from the well-structured and researched international scoping review on RIH by Lehoux et al.^1^ This review is an important contribution to the subject and puts forth the emphasis that stakeholders involved in healthcare innovation should not be short-sighted and focus simply on the innovation as an invention but go beyond this into the realm of RIH that also overcomes system-level challenges of equity and sustainability. This commentary invites readers to also consider innovation strategies that may bring about this as well as look at the guiding principles of health and political systems across countries that may hinder the attainment of innovation diffusion and global equity in the health of populations. Finally, this commentary reiterates the emphasis made by the authors of the need for international policy-oriented fora as learning vehicles on RIH that also address system-level challenges, albeit the need to acknowledge cultural differences. In addition, the public has not only the right for transparency on how equity and sustainability challenges are addressed in innovation decisions, but also the responsibilities to contribute to overcome these challenges.

## Ethical issues

Not applicable.

## Competing interests

Author declares that she has no competing interests.

## Author’s contribution

SCB is the single author of the paper.
